# A Rapid and Simple Multiparameter Assay to Quantify Spike-Specific CD4 and CD8 T Cells after SARS-CoV-2 Vaccination: A Preliminary Report

**DOI:** 10.3390/biomedicines9111576

**Published:** 2021-10-29

**Authors:** Mojtaba Shekarkar Azgomi, Marco Pio La Manna, Giusto Davide Badami, Paolo Ragonese, Antonino Trizzino, Francesco Dieli, Nadia Caccamo

**Affiliations:** 1 Central Laboratory of Advanced Diagnostic and Biomedical Research (CLADIBIOR), University of Palermo, 90127 Palermo, Italy; mojtaba.shekarkarazgomi@unipa.it (M.S.A.); marcopio.lamanna@unipa.it (M.P.L.M.); giustodavide.badami@unipa.it (G.D.B.); francesco.dieli@unipa.it (F.D.); 2 Department of Biomedicine, Neurosciences and Advanced Diagnostic (Bi.N.D.), University of Palermo, 90127 Palermo, Italy; paolo.ragonese@unipa.it; 3 Department of Pediatric Hematology and Oncology, A.R.N.A.S. Civico Di Cristina and Benfratelli Hospital, 90127 Palermo, Italy; triznino@hotmail.com

**Keywords:** Pfizer/BioNTech, memory T cell, CD4^+^, CD8^+^, SARS-CoV-2, cytokines

## Abstract

mRNA and Adenovirus vaccines for COVID-19 are used to induce humoral and cell-mediated immunity, with the aim to generate both SARS-CoV-2 B and T memory cells. In present study, we described a simple assay to detect and quantify Spike-specific CD4^+^ and CD8^+^ T cell responses induced by vaccination in healthy donors and in subjects with B cell compart impairment, in which antibody response is absent due to primary immunodeficiencies or CD20 depleting therapy. We detect and quantified memory T cell immune responses against SARS-CoV-2 evocated by vaccination in both groups, irrespective to the humoral response. Furthermore, we identified TNF-α as the main cytokine produced by T memory cells, after antigen-specific stimulation in vitro, that could be considered, other than IFN-γ, an additional biomarker of induction of T memory cells upon vaccination. Further studies on the vaccine-induced T cell responses could be crucial, not only in healthy people but also in immunocompromised subjects, where antigen specific T cells responses play a protective role against SARS-CoV-2.

## 1. Introduction

After more than 2 years, a syndrome case by severe acute respiratory syndrome coronavirus 2 (SARS-CoV-2) has caused more than 4 million deaths worldwide, and it still is a prevalent outbreak [1]. To date, different licensed vaccines for COVID-19 with different techniques are available, and all presumably induce both humoral and cell-mediated immunity, both of which are required for recovery following SARS-CoV-2 infection. In addition, patients with more severe diseases develop neutralizing antibodies (NAb) that correlate with viral load [2,3]. Still, on the other hand, SARS-CoV-2 can spread from cell to cell without exposure to the extracellular environment [4], limiting the role of NAb in reducing viral spread within the host. For this reason, T cells could be important mediators of the protective host response to SARS-CoV-2 infection. Hence, there is an urgent need to consider the role of SARS-CoV-2-specific T cell immune response after vaccination, analyzing either CD4^+^ T cells, which help B cells for antibody production, or CD8^+^ T cells that kill virus-infected cells [5,6]. Therefore, a simple and rapid assessment of T cell immune response after vaccination remains a challenge. Using flow cytometry, Riou and colleagues described a rapid assay to qualitatively and quantitatively measure SARS-CoV-2-specific CD4^+^ T cell responses in 31 healthcare workers [7]. Despite several studies demonstrating the feasibility of detecting SARS-CoV-2-specific CD4^+^ and CD8^+^ T cells, most of them have been performed in SARS-CoV-2-infected individuals and using different techniques. Still, there is very limited knowledge on similar assays performed after vaccination. Here, we report a rapid (~18 h) method to monitor SARS-CoV-2-specific T cell responses after vaccination independently from NAb blood concentration, which is alsousable in patients with primary or secondary B cell defects.

## 2. Methods

This assay relies on simultaneous expression of three cytokines (IL-2, IFN-γ and TNF-α) after 18 h of stimulation in vitro with a pool of lyophilized peptides, consisting mainly of 15-mer sequences with an 11 amino acid overlap, covering the immunodominant sequence domains of the spike glycoprotein of SARS-CoV-2 (PepTivator^®^ SARS-CoV-2 Prot_S, Miltenyi Biotec, Surrey, UK). The study was approved by the Ethical Committee of the University Hospital. Peripheral blood mononuclear cells (PBMCs) were obtained from *n* = 41 fully vaccinated (mRNA vaccine BNT162b2, Pfizer/BioNTech, Mangonza, Germany) individuals, 15 to 21 days after receiving the second dose vaccine. Asymptomatic subjects negative for SARS-CoV-2 with a real-time PCR test were enrolled in the study.

The enrolled subjects (*n* = 40), were divided into four groups; the first three groups were composed of healthy donors (*n* = 28) with different titers of SARS-CoV-2 NAbs (SARS-CoV-2 Trimeric S IgG) at the time of venipuncture: G1 with a level of NAb < 390 BAU/mL, G2 with 390 < NAb < 1040 BAU/mL, G3 with NAb > 1040 BAU/mL. The fourth group (G4) was composed of patients (*n* = 12) without functional B cells and no NAb in peripheral blood (six patients with multiple sclerosis in therapy with ocrelizumab and six patients with primitive B cell deficiencies). The demographic characteristics of the groups are reported in Table 1 while the specific clinical and therapeutic characteristics of G4 patients are reported in Table 2.

PBMCs were stimulated for 18 h at 37 °C 5% CO_2_ in RPMI1640 complete medium, witha spike-specific peptide pool (1 μg/mL) at 1 × 10^6^ cells/mL. RPMI or ionomycin/PMA were included in each sample as negative or positive controls, respectively. Brefeldin-A (10 μg/mL) was added after 2 h.

After 18 h of stimulation, cells were harvested and stained, first with live/dead marker (Zombie dye, Biolegend San Diego, CA, USA) then with mAb anti-human CD3PerCP-Vio^®^ 700, mAb anti-human CD4 PE-Vio^®^ 770 and mAb anti-human CD8 APC. After surface staining, cells were fixed, permeabilized and stained at room temperature for 30 min with mAb to anti-human IL-2 APC-Vio^®^ 770, mAb anti-human IFN-γ FITC and mAb anti-human TNF-α PE. Samples were acquired on a FACSARIA II flow cytometer (BD Bioscience San Jose CA, USA) and analyzed using FlowJo v10 (BD Bioscience San Jose CA, USA).

**Figure 1:**
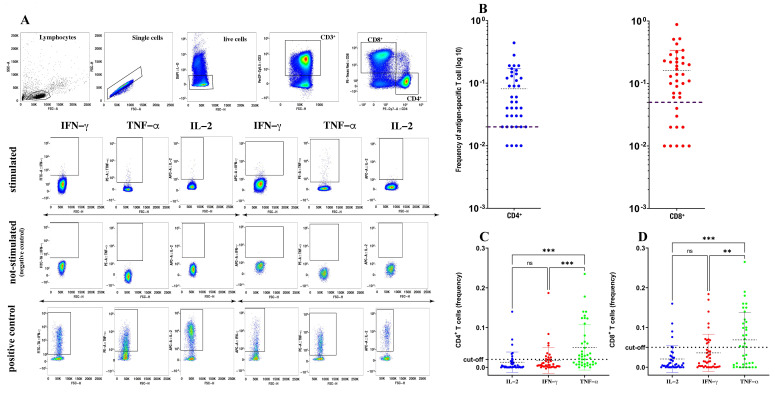
Quantification of spike-specific CD4^+^ and CD8^+^ T cells after SARS-CoV-2 vaccination. Gating strategy used to identify spike-specific CD4^+^ and CD8^+^ T cells and to detect their cytokine expression in response to spike-derived peptides. (**A**) Cumulative frequency and distribution of spike-specific CD4^+^ and CD8^+^ T cells (**B**) in SARS-CoV-2-vaccinated individuals (*n* = 40). Cut-off for positivity was set at <0.02 for CD4^+^ T cells and <0.05 for CD8^+^ T cells. Analysis of distinct cytokine expression by spike-specific CD4^+^ (**C**) and CD8^+^ (**D**) T cells using a Kruskal–Wallis test with Dunn’s correction. ** *p* < 0.01; *** *p* < 0.001; ns: not significant.

The gating strategy is shown in Figure 1A. The threshold for positivity for spike-specific CD4^+^ T cell responses (>0.02%) and antigen-specific CD8^+^ T cell responses (>0.05%) was set according to Dan et al. [7,8,9] and calculated using the median two-fold standard deviation of all negative controls measured. GraphPad software was used to perform statistical analysis, and the groups were analyzed by using a Kruskal–Wallis test with Dunn’s correction and a Pearson correlation test.

## 3. Results

The proportion of vaccinated subjects positive for spike-specific memory CD4^+^ and CD8^+^ T cells, measured as the frequency of CD4^+^ and CD8^+^ T cells simultaneously expressing IL-2, IFN-γ and TNF-α, was 90% (36/40) and 70% (28/40), respectively (Figure 1B). Indeed, both the spike-specific CD4^+^ and CD8^+^ responses were characterized by very faint (if any) IL-2 expression and very low IFN-γ expression, but were enriched in cells expressing TNF-α (Figure 1C,D). Thus, and also in agreement with the study of Riou and colleagues [7], in our assay, TNF-α was the predominant cytokine produced, either by spike-specific CD4^+^ or CD8^+^ T cells, suggesting that TNF-α could be a more reliable biomarker than any other cytokine to detect spike-specific T cells in response to vaccination.

To study the correlation between the spike-specific T cell response and the NAb response, we stratified vaccinated healthy donors into three groups according to the titer of SARS-CoV-2NAbs: G1, <390 BAU/mL; G2, 390–1040 BAU/mL; G3, >1040 BAU/mL.

**Figure 2:**
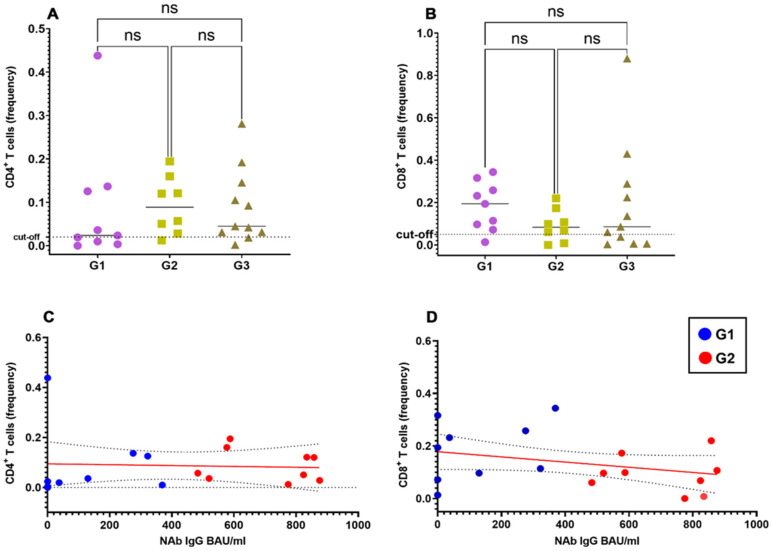
Correlation of spike-specific CD4^+^ and CD8^+^ T cells with spike-specific NAb titers. Frequencies of spike-specific CD4^+^ (**A**) and CD8^+^ (**B**) T cells upon stratification of vaccinated individuals in 3 different groups according to their SARS-CoV-2 NAb titers (groups G1 to G3). Pearson correlation coefficient with one-tailed P-value and 90% confidence interval between titers of SARS-CoV-2 NAbs and the frequency of spike-specific CD4^+^ (**C**) and CD8^+^ (**D**)T cells.

As shown in Figure 2A,B, the spike-specific CD4^+^ and CD8^+^ responses were of similar magnitude irrespective of the NAb titers in the G1, G2 and G3 groups. Accordingly, we did not find significant correlation between frequencies of spike-specific CD4^+^ and CD8^+^ T cells with NAb titers (Figure 2C,D), at least limited to those vaccinated subjects for whom precise antibody titers were available.

Finally, we analyzed the spike-specific T cell response in a group of subjects affected by primary or secondary B cell deficiencies, that had been vaccinated with the Pfizer/BioNTech BNT162b2 mRNA vaccine. Despite failing to produce spike-specific antibodies (Table 3), the B cell-deficient subjects showed a measurable spike-specific T cell response, comparable with that of the other groups.

## 4. Discussion

Much scientific evidence suggests that the cell-mediated response assumes relevant importance for an effective immune response against SARS-CoV-2 virus [10]. One study, in particular, showed that in cancer patients, the establishment of the cell-mediated response induced by the vaccine, even in the absence of a corresponding antibody response, was sufficient to confer protection against infection [11]. Another study has highlighted the establishment of the cellular immune response against SARS-CoV-2 in subjects vaccinated with BNT162b2Pfizer/BioNTech in the absence of an antibody response at the time of the assessment of adaptive immunity, after the second injection [12]. Furthermore, many studies have shown that mRNA vaccines induced a reduced humoral response in patients with acquired immunodeficiencies due to hematological diseases [13] or immunosuppressive therapies, including the use of biological drugs such as rituximab [14,15]. Finally, the Pfizer vaccine also provokeda cell-mediated immune response in subjects with primary immunodeficiencies affecting the B compartment [16]. This literature shows the potential usefulness of a test to evaluate the efficacy of vaccination by measuring the cellular immune response.

Some attempts have been made in this direction, also considering evaluating IFN-γ using IGRA tests [17]. However, the T cells’ response is expressed through different activation pathways, and the evaluation of a single cytokine may not fully reflect the state of activation of this compartment. A multiparametric flow cytometry test, such as the one proposed in this communication, through the evaluation of the simultaneous expression of TNF-α, IL2 and IFN-γ can identify more completely the presence of a cell-mediated immune response induced by vaccination regardless of the evaluation of the humoral response.

Our study shows that vaccination with Pfizer gives a robust cell-mediated immunological memory against spike protein antigens, independently from the titer of NAb, meaning that the T cell-mediated specific immune response against SARS-CoV-2 caused by vaccination can develop independently from B cell response. This finding highlights the importance of the cell-mediated immunity against SARS-CoV-2 induced by vaccination and identifies TNF-α as the main product of T cells of vaccinated subjects after specific stimulation in vitro, making this cytokine a correlate of successful vaccination. In conclusion, we have reported a proof-of-concept study describing a simple and easy assay to detect and quantify spike-specific CD4^+^ and CD8^+^ T cell responses induced by vaccination. Although our study has many limitations (small number of tested individuals, optimization and validation), it provides a tool to monitor the immunogenicity of SARS-CoV-2 vaccines and study the correlation between the quantity and quality of B and T cell-mediated responses and protection. Finally, studying vaccine-induced T cell responses may be of value in those subjects with B cell depletion following ocrelizumab or due to primary immunodeficiencies, in which serological responses are impaired, but T cell responses are preserved.

## Figures and Tables

**Table 1:** Characteristics of enrolled subjects.

	G1 HD, Nab < 390 BAU/mL		G2 HD, 390 < Nab < 1040 BAU/mL		G3 HD, Nab > 1040 BAU/mL		G4 Patients Ocrelizumab or Primitive B Cell Deficiences		Total	
Enrolled Subjects (%)	9	(22.5%)	8	(20%)	11	(27.5%)	12	(30%)	40	(100%)
Mean Age	50		44		45		41		44	
Range	27–66		27–59		22–55		21–64		22–66	
Male Gender (%)	5	(55.7%)	3	(37.5%)	3	(77.5%)	3	(27.3%)	14	(35%)

**Table 2:** Clinical and therapeutic characteristics of G4 enrolled patients.

G4 Enrolled Patients	Pathology	CCI	Therapy	N Days	IgG mg/dL
ID20	RRMS	0	Ocrelizumab	180	-
ID30	RRMS	0	Ocrelizumab	180	-
ID31	RRMS	0	Ocrelizumab	180	-
ID39	RRMS	1	Ocrelizumab	160	-
ID40	SPMS	2	Ocrelizumab	180	-
ID41	RRMS	0	Ocrelizumab	180	-
ID25	CVID	-	IgG Vein Kendrion	28	>600
ID38	CVID	-	Subcutaneous IgG Hyqvia Takeda	28	>600
ID45	XLA	-	Subcutaneous IgG Hyqvia Takeda	28	>600
ID46	CIVD	-	CSL Bhering Hizentra	15	>600
ID47	XLA	-	IgG Vein Kendrion	28	>600
ID48	PNH	-	CSL Bhering Hizentra	15	>600

RRMS = Relapsing–remitting multiple sclerosis;SPMS = Secondary progressive multiple sclerosis; CVID = Common variable immunodeficiency; XLA = Bruton’sagammaglobulinemia;PNH = Paroxysmal nocturnal hemoglobinuria;CCI = Charlson comorbidity index; N days = Days from the last infusion of monoclonal Ab or human IgG with respect to the sampling day.

**Table 3:** NAb titer and frequency of responder T cells in patients with B cell deficiencies.

Enrolled Patients	NAb BAU/mL	Frequency (%) of CD4^+^ T Cells	Frequency (%) of CD8^+^ T Cells
ID20	0	**0.0558**	0.0174
ID25	0	**0.1933**	**0.2069**
ID30	0	**0.0271**	0.0136
ID31	0	**0.0600**	**0.5232**
ID38	0	**0.0350**	0.0256
ID39	0	0.0021	**0.1285**
ID40	0	**0.0390**	0.0230
ID41	0	**0.0867**	**0.0631**
ID45	0	**0.0359**	**0.0969**
ID46	0	0.0120	**0.5109**
ID47	0	**0.1700**	**0.1440**
ID48	0	0.0120	**0.2530**

Values above the cut-off are indicated in bold.

## Data Availability

The Data will be available upon reasonable request to the corresponding author.
