# Correction: Shekarkar Azgomi et al. A Rapid and Simple Multiparameter Assay to Quantify Spike-Specific CD4 and CD8 T Cells after SARS-CoV-2 Vaccination: A Preliminary Report. *Biomedicines* 2021, *9*, 1576

**DOI:** 10.3390/biomedicines11092378

**Published:** 2023-08-25

**Authors:** Mojtaba Shekarkar Azgomi, Marco Pio La Manna, Giusto Davide Badami, Paolo Ragonese, Antonino Trizzino, Francesco Dieli, Nadia Caccamo

**Affiliations:** 1 Central Laboratory of Advanced Diagnostic and Biomedical Research (CLADIBIOR), University of Palermo, 90127 Palermo, Italy; mojtaba.shekarkarazgomi@unipa.it (M.S.A.); marcopio.lamanna@unipa.it (M.P.L.M.); giustodavide.badami@unipa.it (G.D.B.); francesco.dieli@unipa.it (F.D.); 2 Department of Biomedicine, Neurosciences and Advanced Diagnostic (Bi.N.D.), University of Palermo, 90127 Palermo, Italy; paolo.ragonese@unipa.it; 3 Department of Pediatric Hematology and Oncology, A.R.N.A.S. Civico Di Cristina and Benfratelli Hospital, 90127 Palermo, Italy; triznino@hotmail.com

In the original publication, there was a mistake in Figure 1A, as published [1]. A FACS plot for IL-2 production was duplicated during the editing of the Figure through mere error, between non-stimulated and stimulated conditions. In detail, we duplicated the sixth plot of the upper row from the third plot of the middle row. The corrected Figure 1 appears below. The authors state that the scientific conclusions are unaffected. This correction was approved by the Academic Editor. The original publication has also been updated.

## Figures and Tables

**Figure 1:**
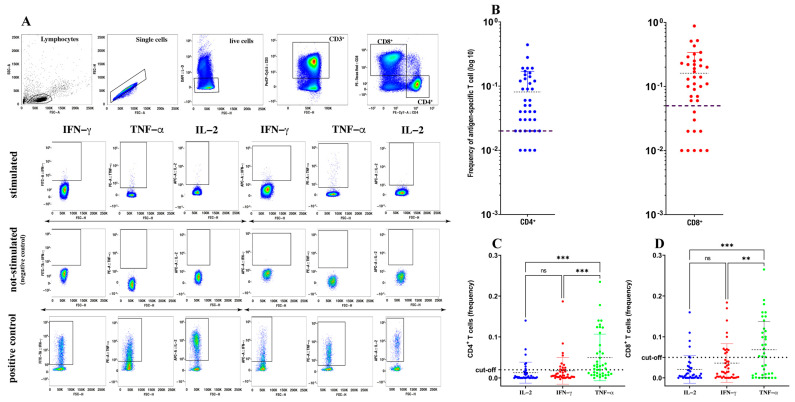
Quantification of spike-specific CD4^+^ and CD8^+^ T cells after SARS-CoV-2 vaccination. Gating strategy used to identify spike-specific CD4^+^ and CD8^+^ T cells and to detect their cytokine expression in response to spike-derived peptides. (**A**) Cumulative frequency and distribution of spike-specific CD4^+^ and CD8^+^ T cells (**B**) in SARS-CoV-2-vaccinated individuals (*n* = 40). Cut-off for positivity was set at <0.02 for CD4^+^ T cells and <0.05 for CD8^+^ T cells. Analysis of distinct cytokine expression by spike-specific CD4^+^ (**C**) and CD8^+^ (**D**) T cells using a Kruskal–Wallis test with Dunn’s correction. ** *p* < 0.01; *** *p* < 0.001; ns: not significant.
